# Systemic inflammatory response syndrome following laparoscopic repair of diaphragmatic injury: A case report

**DOI:** 10.4103/0972-9941.62530

**Published:** 2010

**Authors:** Philip Umman

**Affiliations:** Department of Surgery, T D Medical College, Alappuzha - 688005, Kerala, India

**Keywords:** Diaphragmatic injury, laparoscopic repair, systemic inflammatory response syndrome, tension pneumothorax

## Abstract

Trauma is a major cause of morbidity and mortality in the younger age group. Though diagnostic laparoscopy has been attempted in trauma earlier, with the advance in minimal access techniques, there is an increasing attempt at advancing the indications for laparoscopy in the setting of trauma. Though there are reports and studies on the successful use of laparoscopy in the setting of abdominal trauma, it is essential to remember that laparoscopy in trauma is associated with risks inherent in the procedure itself and also with higher incidence of missed injuries if used as a diagnostic tool.

## INTRODUCTION

Diaphragmatic injuries can occur following both blunt and penetrating abdominal injuries. Approximately 5% of patients with blunt trauma and 10 to 15% of patients with penetrating injuries to the chest and abdomen have diaphragmatic injuries.[1] The overall incidence of diaphragmatic injury is 0.8-5.8% in cases of blunt trauma (2.5-5% in cases of blunt abdominal trauma and 1.5% in cases of blunt thoracic trauma).[2] Penetrating diaphragmatic injuries are more common on the left side, the liver being involved in right-sided injuries. The overall incidence of left-sided diaphragmatic injuries was 42% (59% for gunshot wounds, 32% for stab wounds). In one study, among the 45 patients with diaphragmatic injuries, 31% had no abdominal tenderness, 40% had a normal chest roentgenogram and 49% had an associated haemopneumothorax.[3] Compared to stab injuries, gunshots and other high-energy projectile wounds cause more fragmentation and cavitation resulting in multiple organ injuries and morbidity.[4]

In patients with blunt abdominal trauma who are haemodynamically stable, the trend has shifted towards conservative management if hollow viscus injuries are excluded by a computed tomogram scan of the abdomen. For penetrating injuries, laparotomy was initially considered as the gold standard, but local wound exploration is a useful aid in avoiding unnecessary and negative laparotomies. If the posterior rectus sheath or peritoneum is violated, it is an indication for laparotomy. However, with advances in minimal access techniques, laparoscopy is a viable alternative in selected haemodynamically stable patients. However, laparoscopy in the setting of trauma is associated with risks of missed injuries and problems from the operative procedure itself.

## CASE REPORT

We present the case of a 34-year-old male patient who presented with a stab wound on the anterior abdominal wall. The patient was haemodynamically stable and inspection revealed a 3 cm wound in the left eighth intercostal space just lateral to the mid-clavicular line. Omentum was seen pouting through the wound. There were no other external injuries. In view of the stable haemodynamics, it was decided to do an emergency diagnostic laparoscopy and to proceed to laparotomy if warranted.

**Figure 1:**
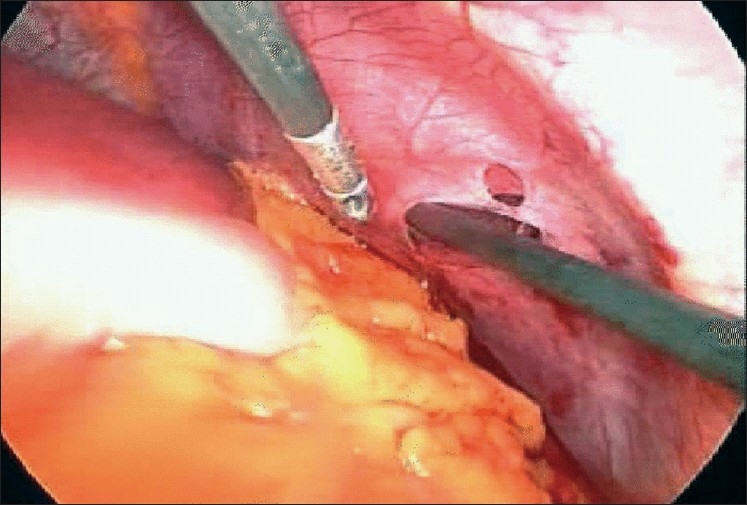
The diaphragmatic tear after the omentum was pulled in to the peritoneal cavity

Diagnostic laparoscopy revealed a 3 cm tear in the apex of the left hemi-diaphragm with omentum pulled in to it and coming out through the skin wound. The spleen, liver, stomach and transverse colon were normal [Figure 1]. There was no haemoperitoneum or free fluid in the peritoneal cavity. There was no hemothorax or lung injury when the scope was introduced into the pleural space after pulling the omentum in to the peritoneal cavity.

### Technique

**Figure 2:**
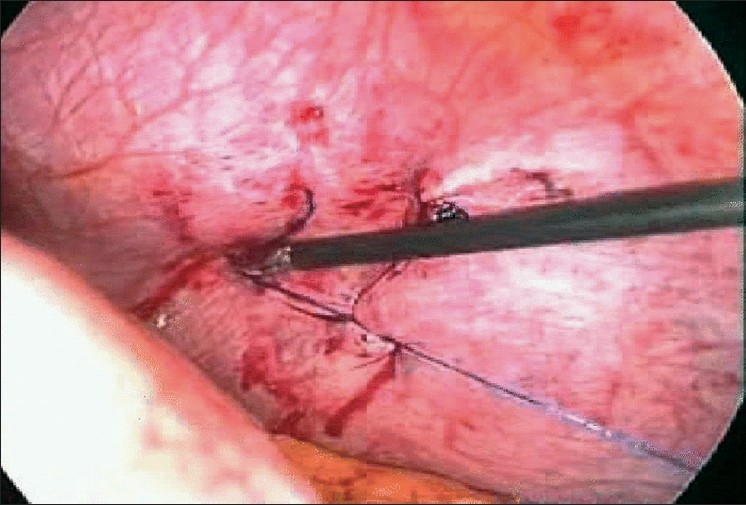
Nearly completed repair of the tear

The primary port (10 mm) was introduced through the umbilicus using the open technique. After confirming the findings, another 10 mm port was put in the left midclavicular line about 6 cm below the subcostal margin and a 5 mm port in the midline in the epigastric area. Once the omentum was pulled down, the patient developed a tension pneumothorax because of air leak from the pneumoperitoneum into the pleural space through the diaphragmatic tear, pushing the left hemi-diaphragm down and obscuring the field. An intercostal drain was introduced in the left fifth intercostal space in the midclavicular line to relieve the pneumothorax. The diaphragmatic tear was sutured using no. 1 polypropylene with intra-corporeal knotting [Figure 2]. At the end of the procedure, a repeat diagnostic laparoscopy was done and then the wounds closed. The patient was started on oral fluids on postoperative day 1. On postoperative day 2, the intercostal drain was removed after confirming complete lung expansion on a chest radiograph.

On day 3, patient developed tachypnoea with fall in oxygen saturation to 90% on room air. There was tachycardia and fever. The patient was shifted to the intensive care unit, started on higher antibiotics and intravenous fluids. Chest radiograph showed patchy infiltrates in both the lung fields suggestive of ARDS. There was fall in the urine output and elevated renal function tests. An ultrasound scan of the abdomen to rule out missed hollow viscus injuries did not show any fluid collection in the peritoneal cavity or pleural space. A diagnosis of systemic inflammatory response syndrome (SIRS) was made, the most probable reason for the same being the sudden tension pneumothorax that developed once the omentum was pulled back from the defect into the peritoneal cavity. The patient recovered with intensive care and non-invasive ventilation and was discharged on the seventh day after surgery. At 1-month follow-up, patient was found doing well and the chest radiograph was normal.

## DISCUSSION

Minimally invasive techniques have been used in the setting of trauma both for diagnosis and treatment. No guidelines are however available for the use of laparoscopy in trauma. The European Association for Endoscopic Surgery has asked for more clinical data on the use of laparoscopy in the setting of trauma.[5] There are case reports of laparoscopic repair of diaphragmatic tears from blunt and penetrating trauma.[6,7] Apart from diaphragmatic tears, hollow viscus perforations have also been tackled by laparoscopy.[8] In patients with wounds on the anterior abdominal wall with omentum herniating through the wound, there are reports of reduction of the omentum followed by diagnostic laparoscopy through the same wound to rule out other associated injuries.[9] There are reports of tension pneumothorax complicating laparoscopy in patients with diaphragmatic injuries. These have been managed with needle thoracentesis or tube thoracostomy.[10] The risk of gas embolism in patients with vascular and liver injuries has also been reported.[11] Patients with concomitant head injury are at high risk of developing intracranial hypertension from laparoscopy.[12]

However, there is a possibility of missing some injuries with a laparoscopy. In an analysis of 37 studies including around 1900 patients where laparoscopy was used as a screening, diagnostic or therapeutic role, it was reported that laparoscopy as a screening tool missed 1% of the injuries and avoided 63% laparotomies. However, as a diagnostic tool, laparoscopy had a 41% to 77% missed injury rate per patient.[13]

## CONCLUSION

Minimally invasive techniques can be used in selected patients with penetrating trauma to the abdomen. However, it is associated with certain risks due to the nature of injury itself apart from those related to the laparoscopy. There are also higher chances of missed injuries, especially of the hollow viscus. A high index of suspicion is necessary in case of clinical deterioration of the patient to prevent mortality from missed injuries after laparoscopy for trauma. The best applications may be in haemodynamically stable patients with stab wounds or tangential wounds to the anterior abdominal wall.[12]
